# Root nodules of red alder (*Alnus rubra*) and sitka alder (*Alnus viridis* ssp*. sinuata*) are inhabited by taxonomically diverse cultivable microbial endophytes

**DOI:** 10.1002/mbo3.1422

**Published:** 2024-06-07

**Authors:** Robyn Dove, Emily R. Wolfe, Nathan U. Stewart, Abigail Coleman, Sara Herrejon Chavez, Daniel J. Ballhorn

**Affiliations:** ^1^ Portland State University Biology Department Portland Oregon USA; ^2^ Portland State University Portland Oregon USA; ^3^ Oregon Health and Science University Portland Oregon USA; ^4^ University of California Berkeley Berkeley California USA

**Keywords:** bacterial endophytes, fungal endophytes, microbial diversity, nitrogen‐fixing symbiosis, phytomicrobiome

## Abstract

The root nodules of actinorhizal plants are home to nitrogen‐fixing bacterial symbionts, known as *Frankia*, along with a small percentage of other microorganisms. These include fungal endophytes and non‐*Frankia* bacteria. The taxonomic and functional diversity of the microbial consortia within these root nodules is not well understood. In this study, we surveyed and analyzed the cultivable, non‐*Frankia* fungal and bacterial endophytes of root nodules from red and Sitka alder trees that grow together. We examined their taxonomic diversity, co‐occurrence, differences between hosts, and potential functional roles. For the first time, we are reporting numerous fungal endophytes of alder root nodules. These include *Sporothrix guttuliformis*, *Fontanospora* sp., *Cadophora melinii*, an unclassified *Cadophora*, *Ilyonectria destructans*, an unclassified *Gibberella*, *Nectria ramulariae*, an unclassified *Trichoderma*, *Mycosphaerella tassiana*, an unclassified *Talaromyces*, *Coniochaeta* sp., and *Sistotrema brinkmanii*. We are also reporting several bacterial genera for the first time: *Collimonas*, *Psychrobacillus*, and *Phyllobacterium*. Additionally, we are reporting the genus *Serratia* for the second time, with the first report having been recently published in 2023. *Pseudomonas* was the most frequently isolated bacterial genus and was found to co‐inhabit individual nodules with both fungi and bacteria. We found that the communities of fungal endophytes differed by host species, while the communities of bacterial endophytes did not.

## Graphical abstract

Plants that host nitrogen‐fixing symbionts in their root nodules play a significant role in both ecology and economy. However, the secondary members of these root nodule microbiomes have not been thoroughly studied. Our field survey conducted on Mount St. Helens revealed that both red Alder and Sitka Alder host a diverse range of cultivable microorganisms in their root nodules.

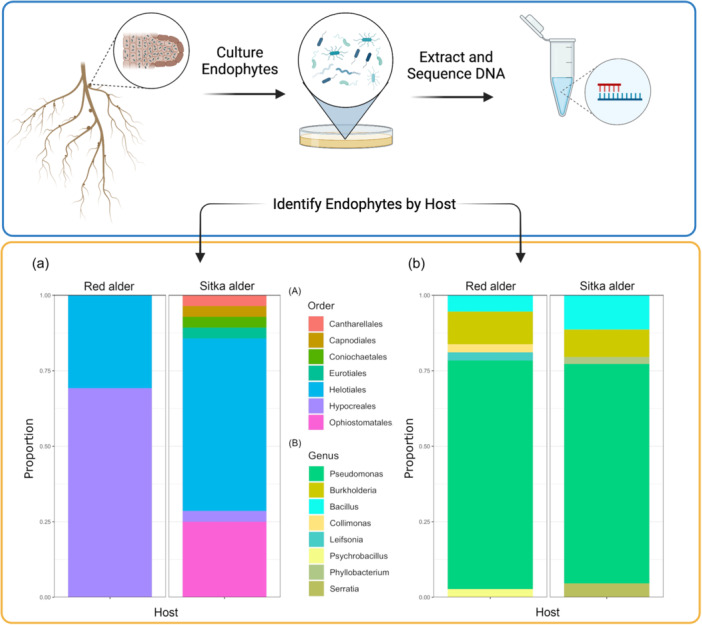

## 1 INTRODUCTION

Symbioses between plants and nitrogen‐fixing bacteria are key drivers of the global nitrogen cycle and can determine the productivity and diversity of terrestrial ecosystems (Galloway et al., 2004; Santi et al., 2013). In addition to supplying their plant hosts with a critical macronutrient (nitrogen), nitrogen‐fixing symbionts (e.g., rhizobia and *Frankia*) can influence plant physiology and impact interactions with plant consumers and higher trophic levels (Antoun et al., 1998; Ballhorn et al., 2013, 2017; Elbadry et al., 2006; Godschalx et al., 2015; Thamer et al., 2011; Vassilev et al., 2006). These root‐nodule‐forming symbioses are most studied in legumes due to their agricultural and ecosystemic importance. Actinorhizal symbioses have received significantly less attention (Schwintzer & Tjepkema, 1990; Tobita et al., 2016; Wolfe et al., 2022a) even though they are equally productive in terms of nitrogen‐fixation rates, contribute as much as 320 kg N ha^−1^ yr^−1^, and provide about 15% of the global plant available nitrogen (Rascio et al., 2008).

Actinorhizal plants include woody plants from eight families that form root nodules with *Frankia*, a group of gram‐positive or gram‐variable filamentous actinomycetes (Benson & Silvester, 1993). Many actinorhizal plants are culturally, ecologically and economically important. For decades, the common understanding was that nitrogen‐fixing bacterial symbionts were the sole inhabitants of root nodules. In recent years, however, taxonomically diverse microbial communities have been found within the root nodules of legumes (Aserse et al., 2013; De Meyer et al., 2015; Deng et al., 2011; Martínez‐Hidalgo & Hirsch, 2017; Rajendran et al., 2012; Velázquez et al., 2013; Wigley et al., 2017) and, in fewer instances, actinorhizal plants (Garneau et al., 2023a; Ghodhbane‐Gtari et al., 2010; Kochkina et al., 2022).

The rhizosphere is rich with microorganisms that interact with plant root exudates and, in turn, can impact plant performance through the production of growth‐enhancing phytohormones and pathogen‐suppressing antibiotics (Breil et al., 1996; Chanway, 2008; Whipps, 2001), as well as through enzymatic mobilization of soil N, P, and S into plant‐available forms (Karagöz et al., 2012; Sharma et al., 2013). Furthermore, Xiao et al. (2017) demonstrated that intranodular microbial communities of soybean (*Glycine max*) and alfalfa (*Medicago sativa*) are distinct from those of the roots and rhizosphere, which supports the idea that the nodule microbiome is selectively curated (Scheublin et al., 2004).

While information on the taxonomic diversity of non‐*Frankia* root nodule endophytes of actinorhizal plants is limited, we know even less about their functional roles in plant physiology and ecosystem processes (but see Garneau et al., 2023a, 2023b). The factors that drive the curation of the root nodule microbiome and determine the diversity and composition of root nodule endophytes also remain elusive, but some studies have shown that the nodule microbiomes of both legumes and actinorhizal plants can be influenced by host genetics, and abiotic factors such as water status (Lipus & Kennedy, 2011; Pozzi et al., 2018; Sharaf et al., 2019). It is well established that edaphic properties can influence soil microbial communities (Lehmann et al., 2011; Zhang et al., 2022) and plant‐microbe interactions (Badri et al., 2009; Wolfe et al., 2022a), but it remains to be seen if edaphic factors also play a role in determining the composition of the actinorhizal root nodule microbiome.

In the Pacific Northwest (PNW), red alder (*Alnus rubra* Bong.) and Sitka alder (*Alnus viridis* [Chaix] DC. ssp*. sinuata* [Regel] A. Löve & D. Löve) are widely distributed actinorhizal trees. In the last few decades, red alder has become one of the most commercially harvested trees in the PNW (Xie, 2008) and the value of its timber has surpassed Douglas fir (*Pseudotsuga menziesii*), the leading lumber tree species in the region. As a result, many red alder plantations have been established in Oregon, Washington and Canada (Tanaka et al., 1997).

Red alder plays a number of important roles in natural habitats; it directly affects the stability and productivity of riparian ecosystems by providing shade and allochthonous inputs (Jackrel & Wootton, 2014, 2015); it enhances soil nitrogen reservoirs (Perakis & Pett‐Ridge, 2019; Teklehaimanot & Mmolotsi, 2007) and it serves as an early colonizer in disturbed ecosystems such as those affected by volcanism, anthropogenic activities, wildfires, landslides, or flooding (Hemstrom & Logan, 1986; Pabst & Spies, 2001). While the smaller, shrub‐like Sitka alder has little or no commercial value as a timber species, it is an important colonizer of disturbed areas at higher elevations with nitrogen‐deficient soils.

In this study, we aimed to characterize the diversity of cultivable, non‐*Frankia* root nodule endophytes in sympatric red and Sitka alder in nature. We chose to isolate cultivable microorganisms, rather than use next‐generation sequencing (which would construct a more complete description of intranodular microbiomes) because cultivable isolates allow for experimental access to the system in the laboratory. Cultivable isolates also have the potential to be used in practical applications in the future. Specifically, we asked 1) How taxonomically diverse are the cultivable non‐*Frankia* root nodule endophytes of red and Sitka alder? 2) Do those endophytes include members that may aid in nitrogen fixation or have other putative beneficial functions? and 3) Do those endophyte communities differ by host?

## 2 MATERIALS AND METHODS

### 2.1 Study area and sample collection

**Figure 1:**
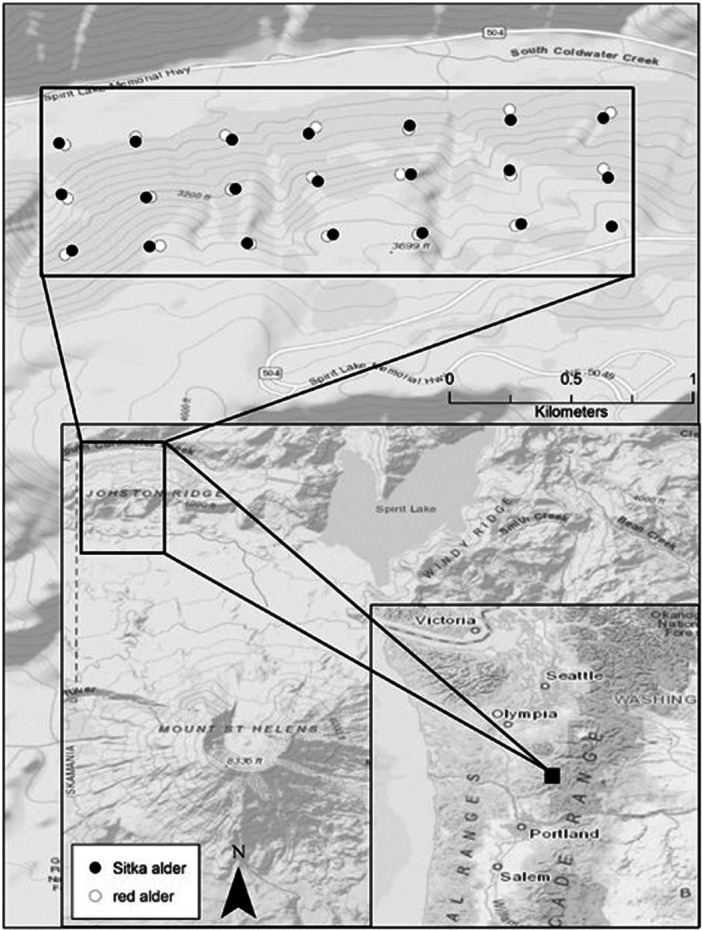
Map of the study area and trees sampled. Red (*n* = 20) and Sitka (*n* = 21) alder trees were sampled from Johnston Ridge, Mount St. Helens (Lawetlat'la), WA.

Root nodules were collected on 5 October 2017, from sympatric red alder and Sitka alder in the Gifford Pinchot National Forest on the northwest side of Lawetlat'la (Cowlitz for Mount St. Helens), Washington, USA (Figure 1). The catastrophic 1980 eruption of Lawetlat'la devastated local ecological communities with landslides, pyroclastic flows, and massive deposits of tephra and ash. The area where our samples were obtained (Johnston Ridge) is located in the lateral blast zone and was left scorched and denuded immediately following the eruption. After four decades of ecological succession, the Johnston Ridge area is still in early succession and is dominated by forbs, including nitrogen‐fixing vegetation such as lupine (Fabaceae: *Lupinus* spp.), and small shrubs like Sitka willow (*Salix sitchensis* Sanson ex Bong.), and alders.

Red and Sitka alder trees were sampled from a sloped area covered with dense, young trees (>15 cm at breast height). The dense, mixed growth enabled the establishment of a very evenly distributed sampling grid as trees of both species grew next to each other throughout the site. Great care was taken to confirm the species and individual tree identity of every root that was selected for root nodule collection. Only roots close to the soil surface that were visibly connected to the individual target tree were considered. Five nodules per tree from a total of 20 red alder and 21 Sitka alder trees were sampled (*n* = 205), collected into sterile falcon tubes, stored in a cooler at 4°C, and processed in the laboratory within 72 h of collection.

### 2.2 Surface sterilization

Nodulated root segments were rinsed with deionized water to remove surface soil and debris before nodules were excised from roots using sterile scalpels and forceps and placed into scintillation vials. Surface sterilization was conducted with a series of 2‐min incubations on a shaker at room temperature (120 rpm at 21°C) and consisted of the following wash steps: two consecutive washes in 5 mL 0.80% NaClO (Clorox brand), followed by three consecutive rinses in 5 mL sterile deionized water. Surface‐sterilized nodules were transferred to sterile 1.5 mL Eppendorf tubes and stored at 4°C until culturing.

### 2.3 Culturing

Single nodules were placed into individual Eppendorf tubes with 200 µL of sterile deionized water, and ground until homogenous with a sterile micropestle. The nodule homogenate was vortexed and centrifuged before 50 µL of the supernatant was pipetted onto lysogeny broth agar (LBA) and malt extract agar with ampicillin (MEA‐AMP) and then spread evenly across the agar using a sterile glass spreader. Inoculated plates were incubated at ambient temperature ( ~ 20°C) until fungal and/or bacterial colonies appeared. Colonies were chosen for subculturing based on morphology, and one colony of each morphotype present on a given plate was subcultured on either LBA (for bacteria) or MEA‐AMP (for fungi) until axenic.

### 2.4 DNA extraction

DNA was extracted from axenic cultures using the Sigma Extract‐N‐Amp kit (Sigma‐Aldrich) with the following modifications to the manufacturer's protocol: small portions (<1 mm^3^) of axenic fungal hyphae or bacterial colonies were placed into 0.2 mL strip‐tubes with 25 μL of extraction buffer using sterile toothpicks; they were then lightly vortexed and heated at 65°C for 10 min, then at 95°C for 10 min, then cooled to 10°C using a BIO‐RAD T100™ thermal cycler; 30 μL of the kit's neutralization solution was added to each sample and the samples were vortexed and centrifuged. The supernatant was reserved as polymerase chain reaction (PCR) template and stored at 4°C.

### 2.5 PCR, sequencing, and operational taxonomic units

To sequence bacterial nodule endophytes, primers 27 F (AGAGTTTGATCMTGGCTCAG) and 1492 R (GGTTACCTTGTTACGACTT) were used on the 16 S rRNA gene (Suzuki & Giovannoni, 1996); primers ITS1F (CTTGGTCATTTAGAGGAAGTAA) and ITS4 (CAGGAGACTTGTACACGGTCCAG) were used to sequence the internal transcribed spacer (ITS) region in fungi (Gardes & Bruns, 1993). The following PCR protocols were used. For 16 S: 95°C (5 min); 35 cycles of 95°C, 45°C, then 72°C (1 min each); 72°C (10 min); and a 4°C hold. For ITS: 94°C (5 min); 35 cycles of 45°C, 50°C, then 72°C (1 min each); 72°C (10 min); and a 4°C hold. PCR reaction mixtures were based on the GoTaq® master mix with the following quantities: 7.75 μL sterile deionized water, 12.5 μL master mix, 1.25 μL BSA, 1.25 μL forward and reverse primers. One μL of 1:10 DNA template was added to the 24 μL reaction mixture. Amplicons were Sanger sequenced in both directions by Functional Biosciences (Madison). Raw forward and reverse reads were visually inspected, trimmed by hand (1400 bp for bacteria and 750 bp for fungi), and assembled using Geneious v10.2.3. Assembly alignments were generated with MAFFT v7.427 via CIPRES/XSEDE (Katoh & Toh, 2010) and then further aligned and clustered into operational taxonomic units (OTUs) at the 99% similarity threshold for fungi (Vu et al., 2019) and 99% threshold for bacteria (Kim et al., 2014) in mothur (Schloss et al., 2009). Bacterial taxonomic assignments were generated by SILVA v132 (Yilmaz et al., 2014) and fungal taxonomic assignments were generated by the UNITE v8 database (Nilsson et al., 2019). Abundance plots were made with the ggplot2 package (Wickham, 2016) in R v4.2.2. The data are openly available at https://github.com/robyndove/AlnusNodules2022. Sequence data can also be found in GenBank at https://www.ncbi.nlm.nih.gov, under accession numbers OK284905 ‐ OK284998 (16 S) and OK338516 ‐ OK338559 (ITS).

### 2.6 Statistics and phylogenetics

**Figure A1:**
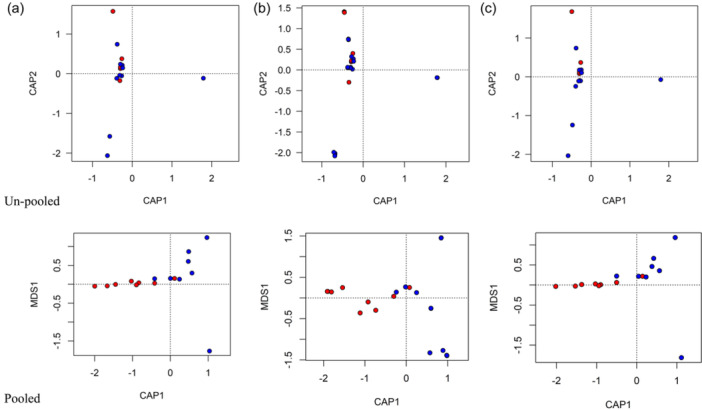
A comparison of distance ordinations for root nodule samples that are un‐pooled versus pooled by tree, using (a) Bray‐Curtis distances, (b) Raup‐Crick distances, and (c) Jaccard distances. Tree species is indicated by dot colour (red =  red alder and blue = Sitka alder).

All statistical analyses were performed using R v4.2.2. Data were analyzed with the “vegan,” and “co‐occur” packages (Griffith et al., 2016; Oksanen et al., 2024). Due to the difficulties inherent in in vitro culturing of rare and/or recalcitrant microbial taxa from root nodules (Gtari et al., 2015), we chose to eliminate the nodules that did not contain cultivable OTUs from all statistical analyses, rather than count them as “truly” absent of fungal or bacterial OTUs. The unique structure of the data matrix (mostly zeros and ones), resulted in the choice to pool nodules by tree individuals and to use relative abundance as the input; this helped to minimize extreme distances in the ordinations (Figure A1), which enabled the use of permutational analysis of variance (PERMANOVA) (Anderson, 2017) using host‐species as the predictor variable in separate analyses of the responses of fungal and bacterial communities. Homogeneity of dispersion among tree species was verified using vegdist, betadisper (bias. adjust=T), and permutest in vegan before proceeding to PERMANOVA with Jaccard distances (Feng et al., 2024) and 999 permutations to determine statistical differences in community composition (Llames et al., 2017). For phylogenetics, representative sequences of each OTU were aligned with downloaded sequences from closely related species (as determined through BLAST searching of the NCBI database) using MAFFT v7.45 (Katoh & Standley, 2013) as implemented in Geneious v10.2.6 (Kearse et al., 2012), followed by manual improvement. Maximum likelihood phylogenies were constructed using RAxML version 8.2.11 (Stamatakis, 2014). For both the ITS and 16 S alignments, GTR + G models were employed, and 500 bootstrap replicates were performed using the rapid bootstrapping algorithm. Phylogenetic trees were visualized with the ggtree package in R (Yu et al., 2017).

## 3 RESULTS

**Figure 2:**
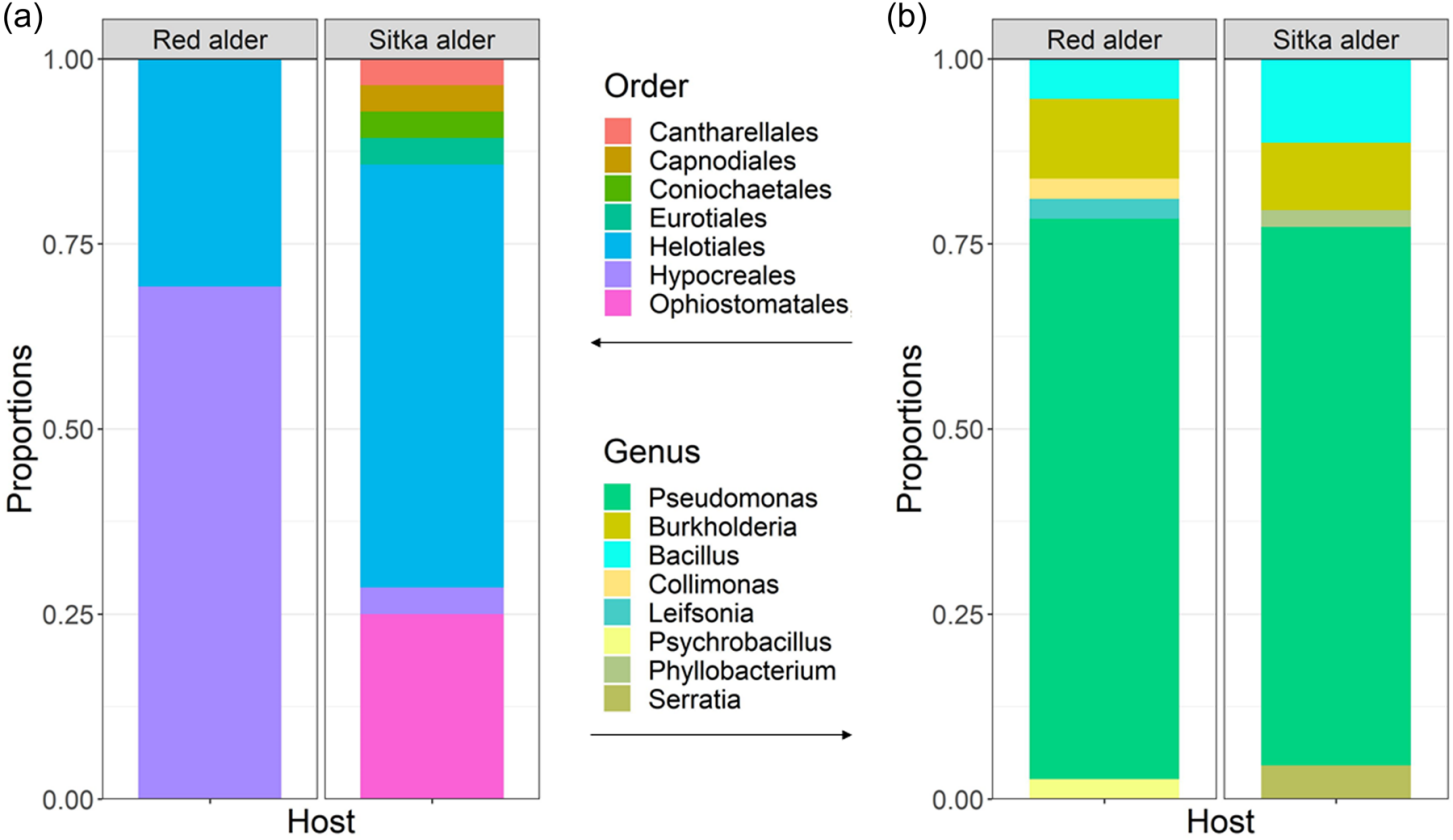
Abundance plots of fungal (a) and bacterial (b) endophytes isolated from root nodules collected in the field, Mount St. Helens (Lawetlat'la), WA. For (A) *n* = 13 red alder root nodules and *n* = 28 Sitka alder root nodules, for (B) *n* = 31 red alder nodules and *n* = 37 Sitka alder root nodules.

We found that cultivable intranodular microbial communities of red alder and Sitka alder are composed of taxonomically diverse endophytic fungi and non‐*Frankia* bacteria (Figure 2). Of the 20 red alder trees sampled, 17 yielded nodules with cultivable operational taxonomic units (OTUs) (average 2.24 ± 0.97 nodules per tree). Of the 21 Sitka alder trees sampled, 19 yielded nodules with cultivable OTUs (average 2.94 ± 1.35 nodules per tree). A total of 205 nodules were collected and 46% yielded cultivable OTUs (red alder = 38 nodules; Sitka alder = 56 nodules; Tables 1 and 2). Of those nodules with cultivable OTUs, 44% (13 red alder and 28 Sitka alder nodules) yielded fungi and 73% (32 red alder and 37 Sitka alder nodules) yielded bacteria. 21% percent of all nodules with cultivable OTUs contained a unique OTU, found only in one nodule (bacterial OTUs 17–25 from 4 red and 5 Sitka alder nodules; fungal OTUs 10, 11, and 13–20 from 1 red and 9 Sitka alder nodules; Table 3).

**Table 1:** Fungal operational taxonomic units and their hosts (R = red alder, S = Sitka alder).

OTU	Phylum	Order	Family	Genus	Species	Host species
1	Ascomycota	Ophiostomatales	Ophiostomataceae	*Sporothrix*	*guttuliformis*	S
2	Ascomycota	Helotiales	Vibrisseaceae			S
3	Ascomycota	Hypocreales	Nectriaceae	*Ilyonectria*	*destructans*	R
4	Ascomycota	Helotiales	Incertae sedis			R, S
5	Ascomycota	Hypocreales	Nectriaceae	*Gibberella*	sp.	R
6	Ascomycota	Helotiales	Nectriaceae	Unclassified		R
7	Ascomycota	Helotiales	Helotiaceae	*Fontanospora*	sp.	R, S
8	Ascomycota	Helotiales	Unclassified			R, S
9	Ascomycota	Helotiales	Helotiaceae	Unclassified		S
10	Ascomycota	Hypocreales	Nectriaceae	*Nectria*	*ramulariae*	R
11	Ascomycota	Helotiales	Incertae sedis	*Cadophora*	*melinii*	S
12	Ascomycota	Helotiales	Incertae sedis	*Cadophora*	unclassified	R, S
13	Ascomycota	Capnodiales	Mycosphaerellaceae	*Mycosphaerella*	*tassiana*	S
14	Ascomycota	Helotiales	Unclassified			S
15	Ascomycota	Hypocreales	Hypocreaceae	*Trichoderma*	unclassified	S
16	Basidiomycota	Cantharellales	Incertae sedis	*Sistotrema*	*brinkmannii*	S
17	Ascomycota	Helotiales	Unclassified			S
18	Ascomycota	Eurotiales	Trichocomaceae	*Talaromyces*	Unclassified	S
19	Ascomycota	Helotiales	Incertae sedis	unclassified		S
20	Ascomycota	Coniochaetales	Coniochaetaceae	*Coniochaeta*	sp.	S

*Note*: When operational taxonomic units (OTUs) could not be identified to a certain taxonomic level, cells were left blank. Rows highlighted in grey indicate OTUs that were isolated from both host species.

**Table 2:** Bacterial operational taxonomic units and their hosts (R = red alder, S = Sitka alder).

OTU	Phylum	Genus	Species	Host species
1	Proteobacteria	*Pseudomonas*	sp.	R, S
2	Proteobacteria	*Pseudomonas*	sp.	R, S
3	Proteobacteria	*Pseudomonas*	sp.	R, S
4	Proteobacteria	*Pseudomonas*	sp.	R, S
5	Proteobacteria	*Pseudomonas*	sp.	R, S
6	Proteobacteria	*Pseudomonas*	sp.	R, S
7	Proteobacteria	*Pseudomonas*	sp.	R, S
8	Firmicutes	*Bacillus*	sp.	R, S
9	Proteobacteria	*Burkholderia*	sp.	R, S
10	Proteobacteria	*Burkholderia*	sp.	R, S
11	Firmicutes	*Bacillus*	sp.	S
12	Proteobacteria	*Pseudomonas*	sp.	S
13	Proteobacteria	*Collimonas*	sp.	R
14	Proteobacteria	*Pseudomonas*	sp.	R, S
15	Proteobacteria	*Pseudomonas*	sp.	R
16	Proteobacteria	*Pseudomonas*	sp.	R
17	Actinobacteria	*Leifsonia*	sp.	R
18	Proteobacteria	*Pseudomonas*	sp.	S
19	Firmicutes	*Psychrobacillus*	sp.	R
20	Proteobacteria	*Pseudomonas*	sp.	R
21	Firmicutes	*Bacillus*	sp.	S
22	Proteobacteria	*Phyllobacterium*	sp.	S
23	Proteobacteria	*Serratia*	sp.	S
24	Proteobacteria	*Burkholderia*	sp.	R
25	Proteobacteria	*Serratia*	sp.	S

*Note*: Rows highlighted in grey indicate operational taxonomic units that were isolated from both red and Sitka alder.

**Table 3:** Co‐occurrence table of the number of times that operational taxonomic units co‐occurred within single root nodules.

Bacterial OTUs	Taxa	Number of occurrences
1 and 10	*Pseudomonas* sp. and *Burkholderia* sp.	1 ^S^
1 and 11	*Pseudomonas* sp. ansd *Bacillus* sp.	1 ^S^
1 and 15	*Pseudomonas* sp. and *Pseudomonas* sp.	1 ^R^
1 and 19	*Pseudomonas* sp. and *Psychrobacillus* sp.	1 ^R^
1, 2, and 8	*Pseudomonas* sp., *Pseudomonas* sp., and *Bacillus* sp.	1 ^S^
1, 2, and 11	*Pseudomonas* sp., *Pseudomonas* sp., and *Bacillus* sp.	1 ^S^
2 and 3	*Pseudomonas* sp. and *Pseudomonas* sp.	2 ^S^
2 and 9	*Pseudomonas* sp. and *Burkholderia* sp.	1 ^S^
2 and10	*Pseudomonas* sp. and *Burkholderia* sp.	1 ^R^
2 and 16	*Pseudomonas* sp. and *Pseudomonas* sp.	1 ^R^
3 and 5	*Pseudomonas* sp. and *Pseudomonas* sp.	1 ^S^
3 and 9	*Pseudomonas* sp. and *Burkholderia* sp.	3 ^R,S^
4 and 13	*Pseudomonas* sp. and *Collimonas* sp.	1 ^R^
4 and 18	*Pseudomonas* sp. and *Pseudomonas* sp.	1 ^S^
4 and 20	*Pseudomonas* sp. and *Pseudomonas* sp.	1 ^R^
6 and 7	*Pseudomonas* sp. and *Pseudomonas* sp.	1 ^R^
8 and 16	*Bacillus* sp. and *Pseudomonas* sp.	1 ^R^

Fungal OTUs	Taxa	Number of occurrences
2 and 7	Vibrisseaceae and Helotiaceae	1 ^S^

Bacterial (B) and Fungal (F) OTUs	Taxa	Number of occurrences
B1 and F2	*Pseudomonas* sp. and Vibrisseaceae	2 ^S^
B1 and F8	*Pseudomonas* sp. and Helotiales	1 ^S^
B2 and F1	*Pseudomonas* sp. and *Sporothrix guttuliformis*	1 ^S^
B2 and F3	*Pseudomonas* sp. and *Ilyonectria destructans*	1 ^R^
B2 and F4	*Pseudomonas* sp. and Helotiales	1 ^R^
B5 and F5	*Pseudomonas* sp. and *Gibberella* sp.	1 ^R^
B5 and F15	*Pseudomonas* sp. and *Trichoderma*	1 ^S^
B6, B7, and F10	*Pseudomonas* sp., *Pseudomonas* sp., and *Nectria ramulariae*	1 ^R^
B7 and F20	*Pseudomonas* sp. and *Coniochaeta* sp	1 ^S^
B15 and F3	*Pseudomonas* sp. and *Ilyonectria destructans*	1 ^R^
B22 and F9	*Pseudomonas* sp. and Helotiaceae	1 ^S^
B24 and F3	*Pseudomonas* sp. and *Ilyonectria destructans*	1 ^R^

*Note*: Each row represents one root nodule. The single row highlighted in grey indicates operational taxonomic units that cooccurred in both red and Sitka alder. Exponents in the number of occurrences indicate host species (R is red alder and S is Sitka alder).

Abbreviation: OTUs, operational taxonomic units.

**Figure 3:**
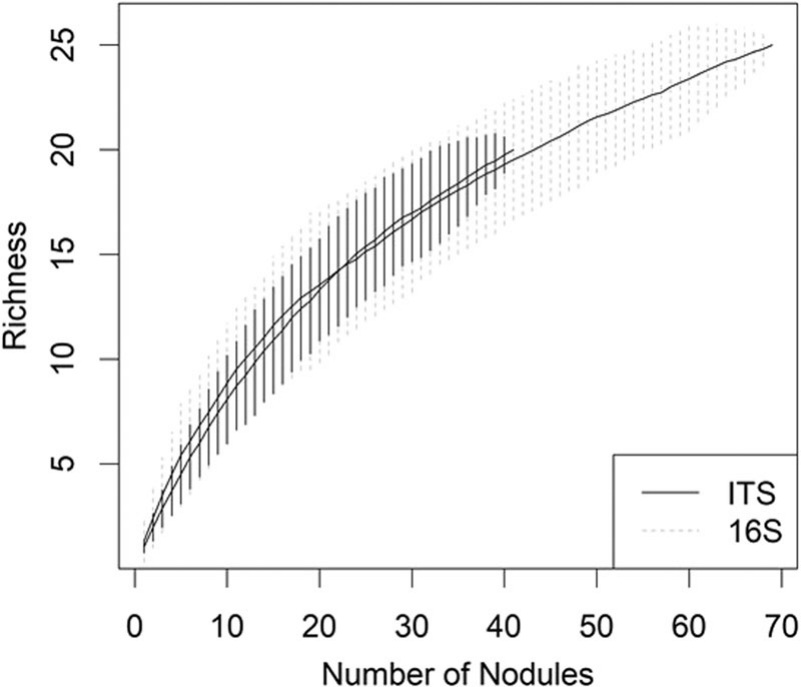
Bacterial (16 S) and fungal (ITS) species richness as a function of number of nodules sampled. Nodules (*n* = 70) were collected from sympatric populations of two alder host species (*Alnus rubra* and *A. viridis*) in a successional environment at Mount St. Helens (Lawetlat'la). Shaded areas represent 95% confidence intervals.

Variation of microbial taxa was observed at the level of the nodule in individual trees (i.e., different nodules on the same tree yielded different cultivable OTUs). Of the 13 red alder trees that had more than one nodule with cultivable endophytes, only one tree had nodules with the same OTU composition. Of the 17 Sitka alder trees, again only one tree had nodules with the same OTU composition. The sampling effort did not exhaust the potential diversity of root nodule endophytes, especially for fungi (Figure 3).

### 3.1 Diversity of cultivable endophytes

**Figure 4:**
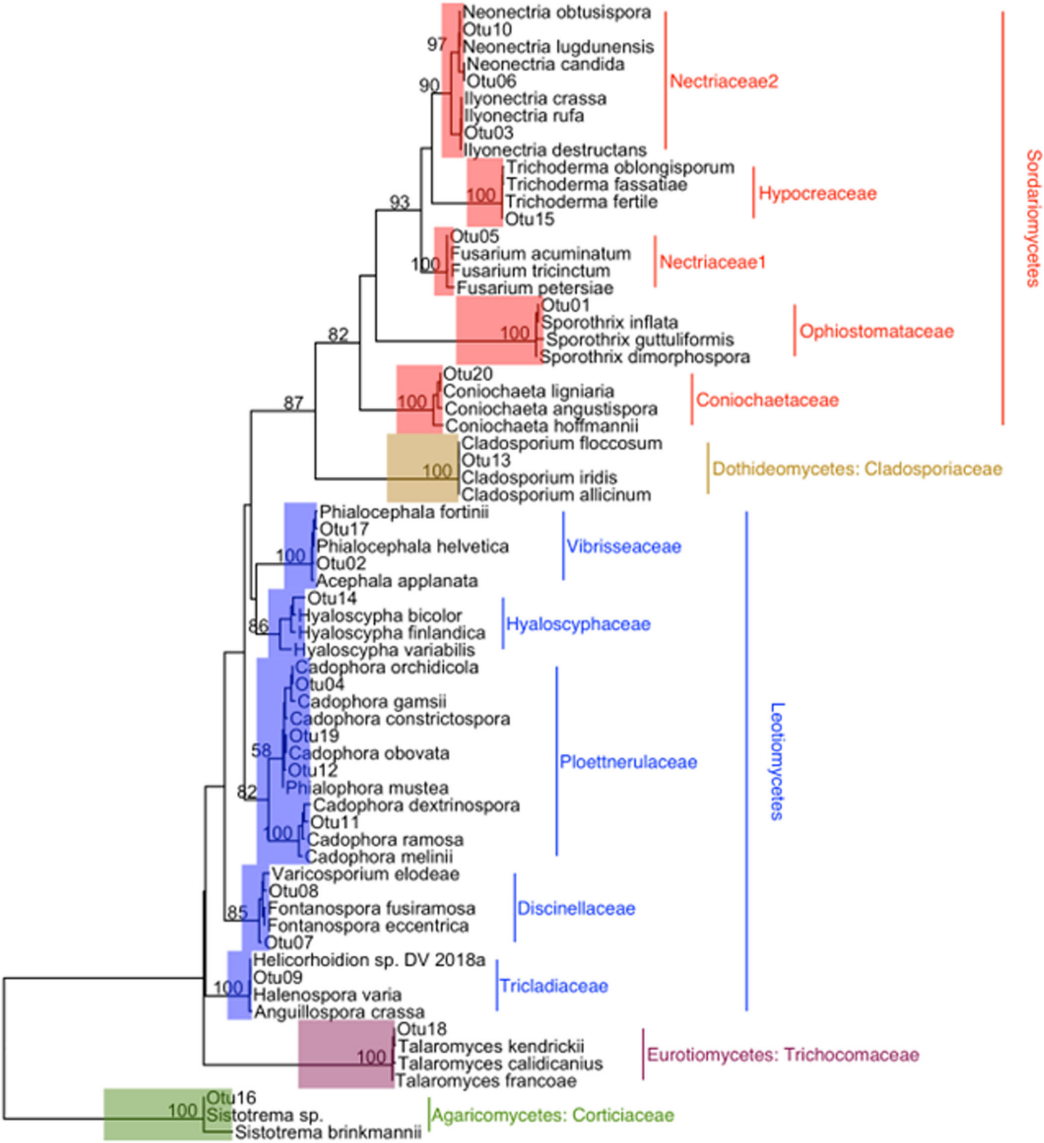
Phylogenetic tree with operational taxonomic units (OTUs) of fungal endophytes, isolated from red and Sitka alder root nodules, placed in the context of closely related taxa. Taxonomic assignments were generated with UNITE v8 in mothur. Numbers at nodes are bootstrap support percentages.

**Figure 5:**
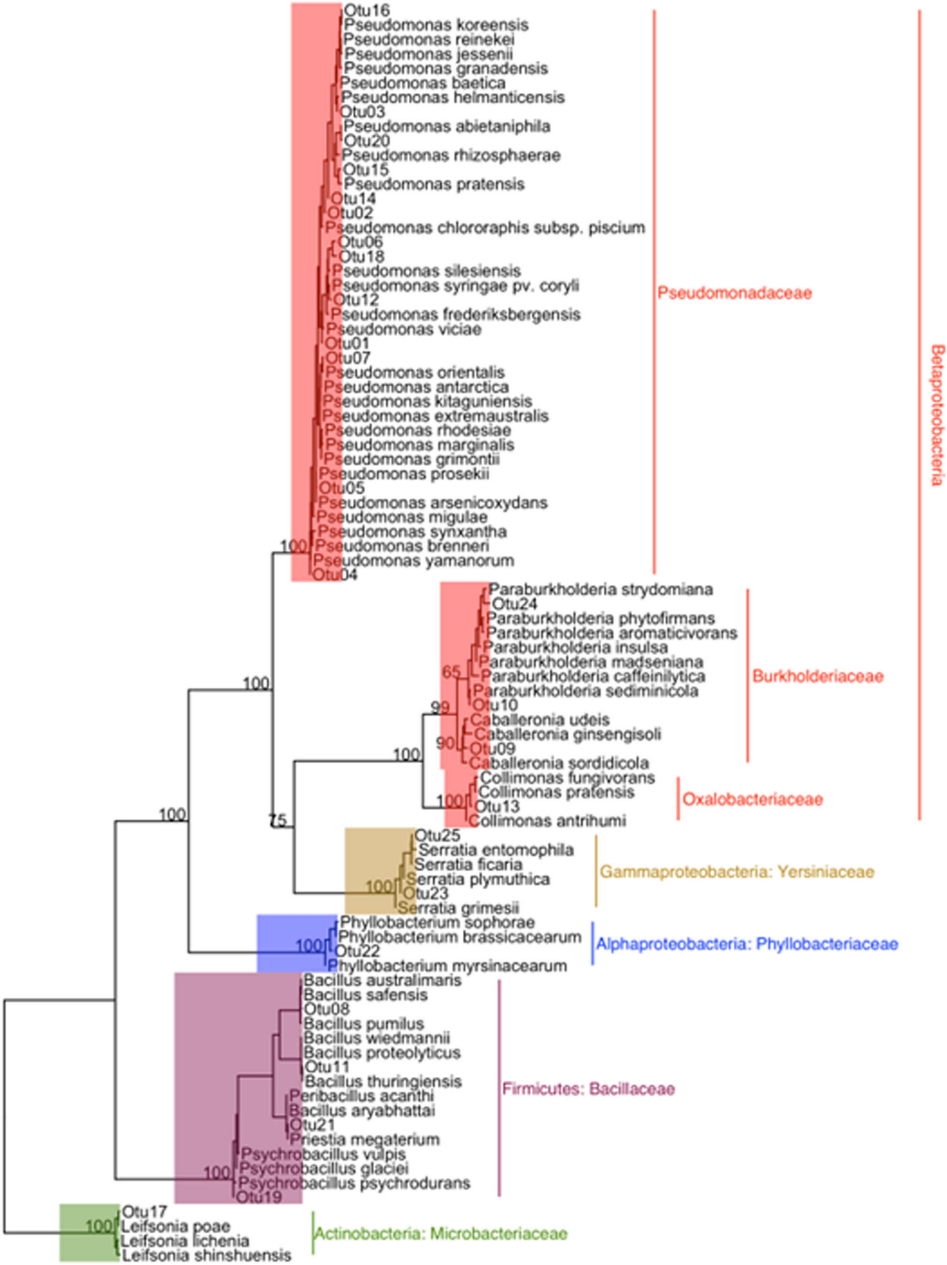
Phylogenetic tree with operational taxonomic units (OTUs) of bacterial endophytes, isolated from red and Sitka alder root nodules, placed in the context of closely related taxa. Taxonomic assignments were generated with SILVA v 132 in mothur. Numbers at nodes are bootstrap support percentages.

A total of 20 fungal OTUs (Table 1) and 25 bacterial OTUs (Table 2) were isolated from nodules of red and Sitka alder. All fungal OTUs belong to phylum Ascomycota (Orders: Ophiostomatales, Helotiales, Hypocreales, Capnodiales, Eurotiales, and Coniochaetales) with one exception (OTU 16 Basidiomycota: *Sistotrema brinkmanii*), isolated from a single Sitka alder nodule (Figure 4). Of the 25 bacterial OTUs, 20 belong to phylum Proteobacteria (*Pseudomona*s sp., *Burkholderia* sp., *Collimonas* sp., *Phyllobacterium* sp., and *Serratia* sp.), 4 belong to Firmicutes (*Bacillus* sp. and *Psychrobacillus* sp.), and one belongs to Actinobacteria (*Leifsonia* sp.) (Figure 5). *Pseudomonas* was the most common and widely distributed genus, representing 13 of the 25 bacterial OTUs, and found in nodules of both red and Sitka alder.

### 3.2 Co‐occurrence of cultivable endophytes

Fungi co‐occurred with other fungi in only one red alder nodule (OTUs 2 and 7: Vibrisseaceae and Helotiaceae). Fungi and bacteria co‐occurred in 17% of nodules (of the 17%, 44% were derived from red alder and 56% from Sitka alder). Bacteria co‐occurred with other bacterial OTUs in 21% of nodules (45% were from red alder and 55% were from Sitka alder).

The most observed pairing of OTUs within a single root nodule was between two strains of *Pseudomonas*, which occurred equally in red and Sitka alder and accounted for 22% of nodules where co‐occurrence was observed. *Pseudomonas* and *Burkholderia* accounted for 18% of all co‐occurrences. *Pseudomonas* and *Bacillus* accounted for 11% of all co‐occurrences. *Collimonas* and *Psychrobacillus* were each found in co‐occurrence with *Pseudomonas* just once and only in red alder. When bacteria and fungi co‐occurred, the bacterial partner was always *Pseudomonas* but the fungal partner varied widely (Table 3). *Pseudomonas* and *Ilyonectria destructans* were the most common pairing of bacterial and fungal endophytes, observed three times—only in red alder—and accounting for 33% of all co‐occurrences of bacteria with fungi.

### 3.3 Host community differences

Fungal endophytes exhibited significant differences in community composition between host species (PERMANOVA: *p* = 0.005; Table 4). Of the fungal OTUs, four were found in nodules of both host species, four were found only in red alder and twelve were found only in Sitka alder. In contrast, bacteria did not exhibit significant differences in community composition between red and Sitka alder hosts. Of the bacterial OTUs, eleven were found in both host species and seven each were found in just red alder or Sitka alder.

**Table 4:** Host effect of bacterial and fungal endophytes isolated from root nodules of red and Sitka alder trees as determined by permutational analysis of variance with Jaccard distances and 999 permutations.

Predictor	Response	F statistic	R^2^	p‐value
Host species	Fungal community	2.223	0.07878	0.005
Host species	Bacterial community	0.706	0.02301	0.828

*Note*: Root nodules were pooled by tree individual and relative abundance of operational taxonomic units was used as the input.

## 4 DISCUSSION

This work demonstrates that root nodules of sympatrically occurring red alder and Sitka alder trees are inhabited by taxonomically diverse cultivable endophytic fungi and non‐*Frankia* bacteria in nature. This corroborates recent work by Garneau et al. (2023a) who were the first to report high levels of microbial diversity from endophytic actinorhizal root nodule cultures of lab‐grown speckled (*Alnus incana* ssp. *rugosa*) and green alder (*A. alnobetula* ssp. *crispa*) seedlings. Also corroborated are McEwan et al. (2015) who used next‐generation sequencing to demonstrate that the endophytic microbial communities of black alder (*A. glutinosa*) root nodules include a small fraction of non‐*Frankia* bacteria. Other studies have shown that actinorhizal plants, including alders, can form multi‐partite associations with arbuscular mycorrhizal fungi, ectomycorrhizal fungi, *Frankia*, and non‐*Frankia* bacteria in root systems (Gardner, 1986; Kennedy et al., 2015; Pozzi et al., 2018; Thiem et al., 2018). However, the taxonomic diversity of cultivable bacteria and fungi ***within root nodules in nature***, reported here, has never been described for any alder species. To our knowledge, this is the first time that any of the fungal isolates sequenced in this study (*Sporothrix guttuliformis*, *Fontanospora* sp., *Cadophora melinii*, *Cadophora* unclassified, *Ilyonectria destructans*, *Gibberella* unclassified, *Nectria ramulariae*, *Trichoderma* unclassified, *Mycosphaerella tassiana*, *Talaromyces* unclassified, *Coniochaeta* sp., and *Sistotrema brinkmanii*) have been reported as root nodule endophytes of alder. We are also reporting, for the first time in alder root nodules, several genera of bacteria (*Collimonas*, *Psychrobacillus* and *Phyllobacterium*).

Our study revealed within‐plant variation of non‐*Frankia* root nodule endophyte composition for both host species. Such nodule‐level variation has been reported for legumes (Mayhood & Mirza, 2021), but, to our knowledge, this is the first time that it has been reported for an actinorhizal plant. Within‐plant variation could imply that the structure of the nodule microbiome is not as tightly controlled by the actinorhizal hostplant as might be expected from studies on legumes (Pang et al., 2021; Sharaf et al., 2019). Instead, community effects (Fukami et al., 2010; Mack & Rudgers, 2008), historical contingency, or stochastic factors might be important drivers of the intranodular microbiome in *Alnus* spp. Conversely, the structure of the root nodule microbiome may be so tightly controlled by the host plant and/or environmental factors that variation occurs at a finer level than that of the whole plant (i.e. at the level of the individual nodule). In our study, we grouped individual nodules by tree for host community analyses (Figure A1). We feel a convincing argument can be made for this being the biologically appropriate level of analysis since we know that host genotype is a major driver of nodule community composition in legumes (Sharaf et al., 2019). Our finding of nodule‐level variation is interesting, however, and the question of “what is the biologically appropriate sampling unit, the nodule or the tree?” should be further explored.

The culture‐based approach employed in this study enables the use of nodule endophytes in future experimentation and, while extensive work still needs to be done, these cultivable isolates have the potential to be developed into inocula for practical applications. While cultivable nodule endophytes hold great promise for manipulative experimentation and the development of biotechnologies, it is important to note the limitations of culture‐based approaches in microbiome studies. Cultivating microorganisms from environmental samples in vitro remains a key challenge in microbial ecology and, in the present study, we recovered culturable OTUs from only 46% of nodules, which led to the question of how to analytically handle the “empty” nodules. Recent evidence from high‐throughput sequencing studies has revealed that actinorhizal root nodules very likely always contain a small fraction of non‐*Frankia* microbial inhabitants (Taş et al., 2023; McEwan et al., 2015). This led us to the decision to exclude the nodules without cultivable endophytes from our statistical analyses rather than to count them as “truly” absent. However, this is not a one‐size‐fits‐all formula for microbial ecology studies since different biological systems will require different treatments. Furthermore, microorganisms that are recalcitrant to laboratory cultivation methodologies, but potentially ecologically important, will go undetected in culture‐based studies. Future studies should employ high‐throughput sequencing of nodule microbial consortia alongside culture‐based approaches to develop a more comprehensive representation of the root nodule microbiome.

### 4.1 Co‐occurrence of cultivable endophytes

This study demonstrates that a single actinorhizal root nodule can be composed of more than one microbial symbiont, which could have functional ramifications for the host plant and ecosystem. Although functional analyses of root nodules with diverse microbial consortia remain sparse, there is ample evidence that microbial interactions in the rhizosphere can impact the health and vigour of nitrogen‐fixing plants. For example, interactions between mycorrhizal fungi and *Frankia* in the rhizosphere of actinorhizal plants can have multiple synergistic effects that benefit the host, including increased biomass (when compared to alder plants grown with just mycorrhizal fungi or *Frankia*), increased leaf N and P content, improved resilience in defoliation experiments, and increased root nodule quantity and size (Oliveira et al., 2005; Struková et al., 1996); Yamanaka et al. (2005) demonstrated that *Alnus sieboldiana* inoculated with *Frankia*, mycorrhizal fungi and *Pseudomonas* exhibited increased biomass as a result of the synergistic interactions of the microbial partners; and Doan et al. (2020) showed the synergistic effects of co‐inoculation of *B. subtilis* and *Collimonas* in controlling Fusarium wilt of tomato and proposed the idea of “biocombicontrol”—the use of two or more strains of beneficial bacteria to control pathogens and enhance plant performance and yield. Considering emerging research on the effects of intranodular microbial interactions on the community structure and metabolic environment of legume root nodules (Hansen et al., 2020) no such information exists for actinorhizal plants), it is very likely that intranodular microbial interactions also influence intranodular diversity and function in actinorhizal systems.

Since many of the fungal strains in this study have known mycotrophic and mycoparasitic lifestyles, and they are being considered as potential biocontrol agents (Carrion & Rico‐Gray, 2002; Cummings et al., 2016; Fukuda et al., 2019), it may not be surprising that we observed the co‐occurrence of fungal strains in only one root nodule. This extremely low degree of co‐occurrence—coupled with the fact that many nodules yielded zero fungal strains—might be attributable to a general lack of endophytic compatibility between hosts and rhizosphere fungi in this system. The low co‐occurrence of fungal strains could also represent an ecological trade‐off for the host wherein the biocontrol and/or nutritive benefits of harbouring more than one strain are outweighed by the costs of maintaining high levels of intranodular diversity (Chen et al., 2020). Alternatively, the observed lack of fungal co‐occurrence might very well be an artefact of the culture‐based methods employed here; it is possible that multiple fungal strains co‐inhabit most, if not all, alder root nodules, but only a limited number of those strains are cultivable, or the non‐cultivable strains exclude the cultivable ones.

When fungi and bacteria co‐occurred in this study, the bacterial partner was always a strain of *Pseudomonas*. Many studies have reported on the synergistic effects of *Pseudomonas* with mycorrhizal fungi and other soil fungi (Sabannavar & Lakshman, 2011; Sreenivasa & Krishnaraj, 1992) and, while speculative, our findings may suggest that *Pseudomonas* functions as a synergistic generalist regarding its multi‐partite associations with plants and other microorganisms. Alternatively, many strains of *Pseudomonas* produce fungicidal secondary metabolites such as proteases, siderophores, and hydrogen cyanide (Ahmadzadeh et al., 2006) so it could be that *Pseudomonas* is playing an antifungal role within root nodules of alders. The latter might explain our finding that *Ilyonectria destructans*—a soil‐borne plant pathogen (Farh et al., 2018)—and *Pseudomonas* were the most common pairing of fungal and bacterial endophytes in this study; certain strains of Pseudomonas may be recruited into nodules in response to infection by *I. destructans*. It is important to note, however, that some pathogenic fungi do not produce disease symptoms when living as endophytes (Chauvet et al., 2016).

We found *Pseudomonas* with *Burkholderia* and *Pseudomonas* with *Bacillus* to be frequent intranodular bacterial pairings. Except for Garneau et al. (2023a, 2023b), almost no other studies have reported on cultivable non‐*Frankia* bacterial endophytes of alder root nodules. In contrast, many studies conducted on legumes have revealed diverse intranodular bacteriomes that include genera such as *Pseudomonas*, *Burkholderia*, and *Bacillus* (Martínez‐Hidalgo & Hirsch, 2017). Future studies will need to test both antagonistic and synergistic activities of intranodular isolates in controlled laboratory and field settings to derive a more complete understanding of the factors that determine the duration of the intranodular microbiome as well as the functional ramifications of interactions among microorganisms.

### 4.2 Putative functional roles of fungal endophytes

The cultivable fungal endophytes isolated in this study comprise a diversity of putative functional roles (Table 5). We isolated taxa within the order Helotiales numerous times in both host species and many of them are unclassified or *incertae sedis*. Plant endophytism is common among members of Helotiales, and fungi within this order have been classified into a variety of ecological niches including mycorrhizae, plant and fungal pathogens, root symbionts, and saprobes (Wang et al., 2006).

**Table 5:** Taxonomic assignments and putative functional roles of fungal endophytes isolated from red and Sitka alder root nodules.

Phylum	Order	Family	Genus species	Host(s)	Putative Function(s)	Literature Cited
Ascomycota	Ophiostomatales	Ophiostomataceae	*Sporothrix guttuliformis*	S	Biocontrol (mycoparasite)	(Carrion & Rico‐Gray (2002))
	Helotiales	Vibrisseaceae	Unclassified	S	Antiinsectan Asymptomatic endophyte	(Fors et al. (2020); Miller et al. (2002); Oehl & Körner (2014))
		Unclassified	Unclassified	R, S	Mycorrhizae Plant pathogen Mycoparasite	(Wang et al. (2006))
		Helotiaceae	*Fontanospora* sp.	R, S	Bioremediation (heavy metal)	(Guimarães‐Soares et al. (2006))
			Unclassified	S	Mycorrhizae Plant pathogen Mycoparasite	(Wang et al. (2006))
		*Incertae sedis*	*Cadophora melinii*	S	Plant pathogen	(Gramaje et al. (2011); Prodi et al. (2008); Travadon et al. (2015))
			*Cadophora* unclassified	R, S	Plant pathogen Asymptomatic endophyte	(Crous et al. (2015); Maciá‐Vicente et al. (2020); Travadon et al. (2015))
	Hypocreales	Nectriaceae	*Ilyonectria destructans*	R	Plant pathogen Biocontrol (antifungal)	(Cabral et al. (2012); Farh et al. (2018); White et al. (1962))
			*Gibberella* unclassified	R	Plant pathogen	(Desjardins (2003))
			unclassified	R	Plant pathogen Bioremediation	(Lombard et al. (2015); Ye et al. (2020))
			*Nectria ramulariae*	R	Plant pathogen	(Hirooka et al. (2012); Tong et al. (2021))
		Hypocreaceae	*Trichoderma* unclassified	S	Bioremediation Mycoparasite Plant growth promotion Biocontrol (antibiotic)	(Cummings et al. (2016))
	Capnodiales	Mycosphaerellaceae	*Mycosphaerella tassiana*	S	Asymptomatic endophyte Plant pathogen	(Bakhshi & Arzanlou (2017); Jalkanen (2016); Petrie & Vanterpool (1978))
	Eurotiales	Trichocomaceae	*Talaromyces* unclassified	S	Mycoparasite Plant growth promotion	(Kato et al. (2012); Shiraishi et al. (2011))
	Coniochaetales	Coniochaetaceae	*Coniochaeta* sp.	S	Plant pathogen	(Damm et al. (2010); Lopez et al. (2007))
Basidiomycota	Cantharellales	*Incertae sedis*	*Sistotrema brinkmannii*	S	Plant pathogen Mycorrhizae Asymptomatic endophyte	(Potvin et al. (2012))

*Note*: Taxonomy is based on 99% similarity and assignments were generated with UNITE v8 in mothur. Eighteen of the twenty operational taxonomic units (OTUs) isolated in our study are represented here. Three OTUs are contained in the Order, Helotiales with Family identified as “unclassified”, and two OTUs that were identified as Family‐Incertae sedis within Helotiales were excluded from this summary.

Taxa within the order Hypocreales were also common among the isolates in this study. They were found primarily in root nodules of red alder, but one isolate was derived from Sitka alder. The Sitka alder isolate (*Trichoderma* unclassified) has been reported in the rhizospheres of young and old alder communities (Sampò et al., 1997) and is a known root endophyte with a range of potential benefits to the host plant including biocontrol properties (Cummings et al., 2016). All Hypocreales isolates from red alder were in the family Nectriaceae, which comprises fungi of diverse functional roles including plant and human pathogens as well as biomedical, biocontrol, and bioremediation agents (Lombard et al., 2015; Ye et al., 2020). Ye et al. (2020) recently discovered a high abundance of Nectriaceae strains in mine‐contaminated soils of Hechi City, China, and speculated that the demonstrated metal tolerance of members of Nectriaceae could implicate this family for use in bioremediation of heavy‐metal contaminated soils.

Our samples were collected in the lateral blast zone of Lawetlat'la and, while the chemical properties of soils in the heterogeneous landscape of post‐eruption Lawetlat'la have not been extensively studied, there is evidence that they contain elevated levels of metals such as magnesium, copper, aluminium, and iron (Dahlgren et al., 1999; Wolfe et al., 2022a). Microorganisms that can tolerate and remediate trace metals are to be expected in post‐eruption volcanic soils (Parelho et al., 2016) but their presence within root nodules of early successional actinorhizal trees is intriguing and begs several questions that our data cannot answer: do these microbes alleviate metal stress for their hosts? Does the host plant “let them in” because of their metal‐tolerant properties, or is their presence inside nodules merely reflective of a scarcity of symbiont options in the rhizosphere? Interestingly, metals such as copper and iron are critical co‐factors to the enzymatic reactions that drive biological nitrogen fixation (González‐Guerrero et al., 2014); the metal‐tolerant fungi within root nodules could potentially facilitate intranodular N_2_‐fixation by mobilization of metal co‐factors. These questions warrant further investigation as they could be pivotal to the development of bioremediation technologies involving actinorhizal plants. For example, Lalancette et al. (2019), explored the use of *Alnus* species, along with their fungal endophytes, to stabilize metal‐contaminated sites. They demonstrated that certain fungal endophytes from alder, when used as soil inoculants, can promote plant growth even under metal stress conditions.

Notably, many of our fungal taxa were only isolated from Sitka alder nodules. Sitka alder is the dominant tree species in the study area, and it has likely been established there for a longer period than red alder (Titus et al., 1998; Titus, 2008; Wolfe et al., 2022a). Furthermore, the study site is on a north‐facing slope and as such, rather than being left entirely barren in the wake of the eruption's lateral blast, it was denuded of aboveground plant parts; Sitka alder rootstocks and fungal propagules may have survived the blast. Recently, Wolfe et al. (2022b) determined that foliar fungal endophyte communities of woody species (including Sitka alder) on the pumice plane of Lawetlat'la seem to be in the early stages of community development, which could also be the case for root nodule endophytes of *Alnus* spp. found in the same area. The elevated fungal diversity seen in Sitka alder in this study could simply be a function of time since establishment, or there could be other molecular mechanisms at work among Sitka alder and its intranodular microbes that favour promiscuous fungal colonization.

### 4.3 Putative functional roles of bacterial endophytes

The bacterial isolates in this study comprise many putative functional roles, with plant growth promotion being a common theme (Table 6). *Pseudomonas* was appreciably the most common bacterial genus isolated; it was found to occur “alone” as well as together with other cultivable nodule endophytes. *Pseudomonas* is well established as a plant growth‐promoting bacteria in a wide variety of plants including legumes (Cardoso et al., 2018; Sánchez et al., 2014). In addition to growth promotion, some strains of *Pseudomonas* can provide extra nitrogen to plants via independent nitrogen‐fixation (Krotzky & Werner, 1987), while other strains have been shown to improve symbioses with rhizobia and *Frankia* when co‐inoculated (Egamberdieva et al., 2010; Fox et al., 2011; Ibáñez et al., 2009; Knowlton & Dawson, 1983). Under certain circumstances, *Pseudomona*s can form symbiotic root nodules on legumes (Shiraishi et al., 2010).

**Table 6:** Taxonomic assignments and putative functional roles of bacterial endophytes isolated from red (R) and Sitka (S) alder root nodules.

Phylum	Order	Family	Genus	Host(s)	Putative Function(s)	Literature Cited
Proteobacteria	Pseudomonadales	Pseudomonadaceae	*Pseudomonas*	R, S	Plant growth promotion Nitrogen‐fixation Synergism with rhizobia and *Frankia* Nodulating symbiont Biocontrol	(Ahmadzadeh et al. (2006); Cardoso et al. (2018); Fox et al. (2011); Haas & Défago (2005); Ibáñez et al. (2009); Knowlton & Dawson (1983); Knowlton et al. (1980); Krotzky & Werner (1987); Pramanik et al. (2021); Sánchez et al. (2014); Shiraishi et al. (2010))
	Betaproteobacteriales	Burkholderiaceae	*Burkholderia*	R, S	Plant growth promotion Nitrogen‐fixation Synergism with rhizobia and *Frankia* Biocontrol Improve nutrient acquisition	(Barka et al. (2000); Castanheira et al. (2016); Gyaneshwar et al. (2011); Martínez‐Aguilar et al. (2013); Zhang et al. (2021))
			*Collimonas*	R	Biocontrol	(de Boer et al. (2004); Höppener‐Ogawa et al. (2009))
	Rhizobiales	Rhizobiaceae	*Phyllobacterium*	S	Plant growth promotion Rhizobia: nodulation and nitrogen‐fixation	(De Meyer et al. (2015); Flores‐Félix et al. (2015); Rojas et al. (2001); Valverde et al. (2005))
	Enterobacteriales	Enterobacteriaceae	*Serratia*	S	Plant growth promotion Nitrogen‐fixation Synergism with rhizobia Biocontrol Heavy metal tolerance	(Balachandar et al. (2006); Barretti et al. (2009); Khan et al. (2015); Müller et al. (2013); Tavares et al. (2018))
Firmicutes	Bacillales	Bacillaceae	*Bacillus*	R, S	Plant growth promotion Synergism with rhizobia Enhance iron availability Salt tolerance Heavy metal tolerance Biocontrol	(Babu et al. (2013); Doan et al. (2020); Egamberdieva et al. (2017); Gutiérrez‐Mañero et al. (2001); Rajendran et al. (2012))
		Planococcaceae	*Psychrobacillus*	R	Biocontrol Nitrogen‐fixation	(Das et al. (2017); Rilling et al. (2018); Xu et al. (2018))
Actinobacteria	Micrococcales	Microbacteriaceae	*Leifsonia*	R	Plant growth promotion Biocontrol Oxidative stress tolerance Plant pathogen	(Battu & Ulaganathan (2020); Liaqat & Eltem (2016); Monteiro‐Vitorello et al. (2013))

*Note*: Taxonomy is based on 99% similarity and assignments were generated with SILVA v132 in mothur.

*Burkholderia* was the second most common Proteobacteria genus in this study and it was found in root nodules of both red and Sitka alder. *Burkholderia* have been identified within alder root nodules only once before (McEwan et al., 2015), but they are common in legume root nodules where they can promote plant growth (Castanheira et al., 2016), fix nitrogen (Martínez‐Aguilar et al., 2013), induce nodulation (Gyaneshwar et al., 2011), and act as biocontrol agents (Barka et al., 2000).

Our most common bacterial isolates from within the phylum Firmicutes were strains of *Bacillus*. Plant‐growth‐promoting *Bacillus* are known endophytes of rhizobial root nodules (Li et al., 2008; Sturz et al., 1997) and have also been isolated from roots—but not nodules—of actinorhizal *A. firma* (Shin et al., 2012). Gutiérrez‐Mañero et al. (2001) showed that *B. pumilus* and *B. licheniformis* have considerable growth promotion activity in *A. glutinosa* via the production of bioactive gibberellins, while Babu et al. (2013) demonstrated that a strain of *B. thuringiensis* isolated from roots of *A. firma* increased heavy‐metal uptake in seedlings.

We already know that *Pseudomonas*, *Burkholderia*, and *Bacillus* can be critical to the induction or promotion of nodulation in some legumes and actinorhizal plants (Gyaneshwar et al., 2011; Knowlton et al., 1980; Sturz et al., 1997), but we do not know if their presence within our sampled nodules is an artefact of that initial relationship, or if they enter and persist within nodules as commensals or to serve other roles.

### 4.4 Host community differences

Due to the specific nature of *Alnus* spp. and their mycorrhizal fungi (Molina, 1981), our finding that cultivable fungal endophyte communities also differ between host species is not surprising. Although we did not focus on *Frankia* in this study, the fact that bacterial endophyte communities did not differ by host aligns with findings by Balkan et al. (2020) that *A. rubra* and *A. rhombifolia* lack strong host specificity regarding their *Frankia* mutualists, implicating potential general cross‐compatibility of bacterial symbionts within *Alnus*.

The degree of specificity between a host and its microbiome has broad implications for the ecology and evolution of both host and symbiont (Foster et al., 2017). High host‐specificity, for example, could result in greater symbiotic efficiency (e.g. higher rates of N_2_‐fixation) (Porter & Sachs, 2020), but could also render the system more vulnerable to perturbations due to a lack of diversity and functional redundancy. While low specificity, or high generalism, in host‐microbe systems can enhance host resilience under stress (Petipas et al., 2020), low specificity can also lead to decreased symbiotic effectiveness, since relaxed partner choice can result in less desirable partners.

## 5 CONCLUSIONS

The potentially vast and nuanced functional roles of the microbial isolates identified in this study warrant further research. Such research will be pivotal to developing and improving phytostabilization, bioremediation, and agricultural biotechnologies involving actinorhizal plants. Furthermore, elucidating the diversity, functional roles, and potential host‐specificity of endophytes in the intranodular microbiome will be foundational to our understanding of the mechanisms by which multi‐partite plant‐endophyte relationships establish and persist.

## AUTHOR CONTRIBUTIONS

**Robyn Dove**: Formal analysis (supporting); writing—original draft (lead); writing—review and editing (lead). **Emily R. Wolfe**: Formal analysis (lead); visualization (lead), writing—review and editing (supporting). **Nathan U. Stewart**: Data curation (supporting); formal analysis (Supporting); investigation (supporting); methodology (supporting); visualization (supporting); writing—review and editing (supporting). **Abigail Coleman**: Investigation (supporting); methodology (supporting). **Sara Herrejon Chavez**: Investigation (supporting); methodology (supporting). **Daniel J. Ballhorn**: Conceptualization (lead); investigation (Supporting); project administration (lead); resources (lead); supervision (lead); writing—review and editing (supporting).

## CONFLICT OF INTEREST STATEMENT

The authors declare no conflict of interest.

## ETHICS STATEMENT

None required.

## Data Availability

All of the data are openly available at https://github.com/robyndove/AlnusNodules2022. Sequence data are also available in GenBank at https://www.ncbi.nlm.nih.gov, under accession numbers OK284905‐OK284998 (16S) and OK338516‐OK338559 (ITS).
